# Recent Advances in the Molecular Pathogenesis of Dystonia-Plus Syndromes and Heredodegenerative Dystonias

**DOI:** 10.2174/157015913804999432

**Published:** 2013-01

**Authors:** Catharina Casper, Eirini Kalliolia, Thomas T Warner

**Affiliations:** Department of Clinical Neurosciences, UCL Institute of Neurology, Royal Free Campus, Rowland Hill Street, London NW3 2PF, United Kingdom

**Keywords:** Dystonia- plus syndrome, Secondary dystonia, Molecular pathogenesis, Neurodegeneration, Mitochondria.

## Abstract

The majority of studies investigating the molecular pathogenesis and cell biology underlying dystonia have been performed in individuals with primary dystonia. This includes monogenic forms such as *DYT1*and* DYT6* dystonia, and primary focal dystonia which is likely to be multifactorial in origin. In recent years there has been renewed interest in non-primary forms of dystonia including the dystonia-plus syndromes and heredodegenerative disorders. These are caused by a variety of genetic mutations and their study has contributed to our understanding of the neuronal dysfunction that leads to dystonia These findings have reinforced themes identified from study of primary dystonia including abnormal dopaminergic signalling, cellular trafficking and mitochondrial function. In this review we highlight recent advances in the understanding of the dystonia-plus syndromes and heredodegenerative dystonias.

## INTRODUCTION

The dystonias are a heterogeneous group of hyperkinetic movement disorders characterised by involuntary sustained muscle contractions leading to abnormal postures and repetitive movements. The heterogeneity of the disease phenotype has led to difficulties in both the diagnosis and classification of the dystonias. The best-described and most common form of dystonia is primary torsion dystonia (PTD), where dystonia is the key clinical feature, sometimes associated with tremor. PTD is often genetic in origin and there is no evidence of neurodegeneration. The classification and pathogenesis of primary dystonia has recently been reviewed [1].

However, dystonia may also manifest in combination with other neurological features such as parkinsonism or myoclonus in the so-called “Dystonia-Plus” syndromes. These syndromes are also often genetic, again with no evidence of neurodegeneration. Both PTD and Dystonia-Plus syndromes have dystonia as the primary clinical feature. However, a dystonic phenotype may also arise as a consequence of other disorders and is typically associated with additional neurological and non-neurological features. In these conditions, dystonia is a downstream consequence of another disease, and is referred to as secondary dystonia. Some of these are due to genetic degenerative conditions and are subgrouped into heredodegenerative disorders. An increasing number of novel disease genes have been found associated with Dystonia-Plus syndromes and secondary dystonias. Linking genetic mutations to cellular pathogenesis has been problematic, but in recent years the development of cell and mouse models has allowed the delineation of molecular mechanisms and identification of themes underlying of a number of these conditions. In this review we describe recent key advances in understanding molecular and cellular mechanisms of dystonia-plus syndromes and heredodegenerative dystonias.

## DYSTONIA-PLUS SYNDROMES

In this group of conditions, dystonia is associated with another movement disorder, for example myoclonus or parkinsonism. There is no neuronal death and there is a functional neurochemical or neurophysiological defect. Three main syndromes are recognised: dopa-responsive dystonia, myoclonus-dystonia syndrome and rapid-onset dystonia parkinsonism and these are summarised in Table **1**.

## DOPA-RESPONSIVE DYSTONIA (DRD)

### Clinical Phenotype

DRD is a rare form of dystonia. The classic phenotype is of early-onset lower-limb dystonia causing gait disturbance, with diurnal fluctuation and an excellent response to levodopa [2]. Parkinsonism can develop later and be an early feature in adult onset cases. Diurnal fluctuation describes worsening of symptoms toward the evening and improvement after sleep. Other features include brisk reflexes and extensor plantar responses. DRD shows a dramatic and sustained response to small doses of L-dopa (as low as 50 - 200 mg), usually apparent within days to weeks. Motor complications of L-dopa treatment, seen in Parkinson’s disease, rarely develop. Association with anxiety, depression, obsessive-compulsive disorder, and/or sleep disturbances have been reported [3].

### Molecular Pathogenesis

**Fig. (1):**
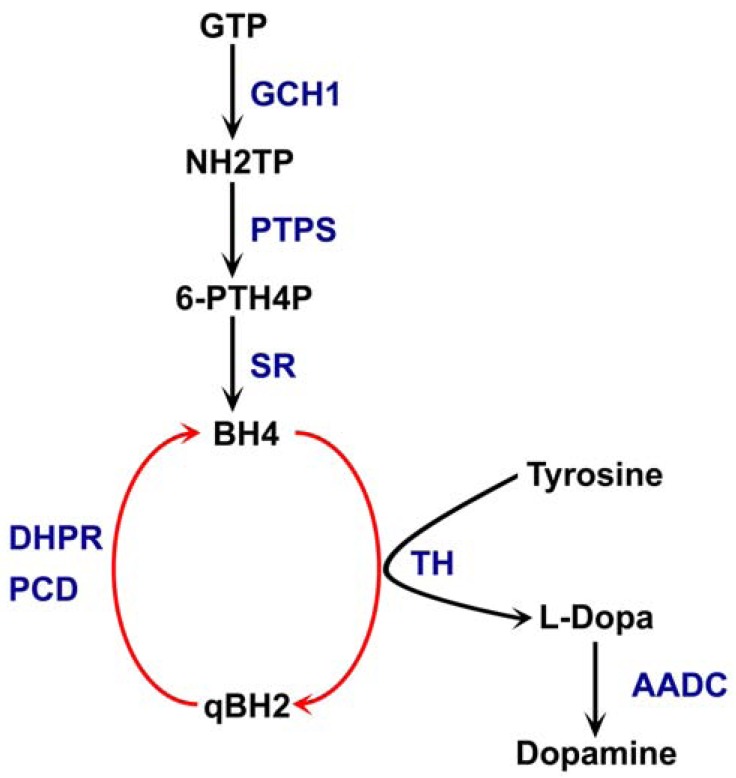
**he biosynthetic pathway of dopamine.** Tetrahydrobiopeterin (BH4) is generated from Guanosine triphosphate (GTP) by consecutive catalytic steps involving the enzymes GTP cyclohydrolase (GCH1), 6-pyrovoyl-tetrahydropetrin synthase (PTPS) and sepiapterin reductase (SR). A pool of BH4 is maintained by the action of Dihydropterine reductase (DHPR) and carbinaloamine-4a-dehydratase (PCD). Tyrosine Hydroxylase (TH) is dependent on BH4 to catalyze the first step in the biosynthesis of dopamine and Aromatic L-amino acid decarboxylase (AADC) generates Dopamine from its precursor L-dopa. Mutations in any of the underlined enzymes have been linked to DRD.

Most cases are autosomal dominant due to heterozygous mutations in the GCH1gene, encoding GTP cyclohydrolase 1. This enzyme catalyzes the biosynthesis of tetrahydobiopterin (BH4), an essential cofactor for tyrosine hydroxylase, the rate-limiting enzyme in the biosynthesis of dopamine (Fig. **1**). BH4 is also a cofactor for noradrenaline and serotonin synthesis, as well as for the enzyme nitrous oxide synthetase. Recessive forms of DRD have also been identified with mutations in the genes for tyrosine hydroxylase (TH), 6-pyruvoyl-tetrahydropterin synthase (PTPS), sepiapterin reductase (SR), dihydropteridine reductase (DHPR) and aromatic acid decarboxylase (AADC). These forms usually have a more complicated and severe phenotype [4].

Patients with DRD have selective striatonigral dopamine deficiency [5] without neuronal loss [6]. The genetic pathogenesis of GCH1mutations has been best studied. Numerous mutations causing missense, nonsense, or frame-shift changes in the GCH1 gene have been described, which appear to produce haploinsufficiency and reduced GCH1 enzyme activity. There has also been a recent report of the first intragenic exon-duplication mutation in the GCH1gene in a DRD family [7].

The predominant and most obvious way by which mutations in GCH1, and other genes, affect dopaminergic neurotransmission is by disrupting the biosynthesis of dopamine. However, a recent study highlighted the potential role of Reactive Oxygen Species (ROS)as a consequence of BH4 deficiency [8]. A delicate balance of tetrahydrobiopterin is crucial for the maintenance of the oxidative environment within cells through its involvement in the nitric oxide synthase system. Failure of this system results in the production of large amounts of free radicals by the mitochondrial electron transport chain and decreases in the cellular production of ATP. This energy depletion and oxidative stress may contribute to the failure of the dopamine system in DRD. Thus the underlying dopaminergic defect may be more complex than simple haploinsufficiency of key enzymes.

## MYOCLONUS-DYSTONIA SYNDROME (MDS; DYT11)

### Clinical Phenotype

The most prominent feature of DYT11 is non-epileptic myoclonus of subcortical origin [9] which manifests as brief lightning jerks movements affecting the neck, trunk, and upper limbs [10]. Legs are less prominently affected. It has an estimated prevalence of about 2 per million in Europe. Approximately 50% of affected individuals have additional focal or segmental dystonia, presenting as cervical dystonia and/or writer's cramp. Non-motor features may include obsessive-compulsive disorder (OCD), depression, anxiety, personality disorders, alcohol abuse, and panic attacks.

Symptom onset is usually in childhood or early adolescence but ranges from age six months to 80 years. Most affected adults report a dramatic reduction in myoclonus in response to alcohol ingestion. Treatment with benzodiazepines can be helpful, although habituation is common. One study of bilateral globus pallidum internus deep brain stimulation (DBS) in *DYT11*MDS found it to be safe and highly effective [11]. The median myoclonus score and dystonia score decreased significantly after DBS, disability was improved and symptoms remained stable between the postoperative evaluations. No adverse effects occurred. This therapeutic option could therefore be considered for patients with severe, drug-resistant forms of MDS. In spite of these encouraging reports, the mechanism by which DBS improves symptoms in MDS is largely unknown.

### Molecular Pathogenesis

MDS is inherited as an autosomal dominant trait with reduced penetrance due to maternal genomic imprinting. Mutations have been identified in the epsilon-sarcoglycan gene (SGCE, DYT11) in many individuals [12]. In humans, almost exclusively paternal SGCE alleles are transcribed, whereas maternal alleles appear to be inactivated by promoter methylation [13,14]. Consequently, clinical symptoms of DYT11 are absent in over 95% of patients who inherited a SGCE mutation from their mother. In these patients, the inheritance pattern may appear pseudo-recessive or pseudo-sporadic [15].

Sarcoglycans are transmembrane proteins that are part of the dystrophin-associated glycoprotein in cardiac and skeletal muscle. In the brain, SGCE is found in midbrain monaminergic neurons, cerebellar Purkinje cells, hippocampus and cortex [16]. To date little is known about the precise cellular role of ε-sarcoglycan in neurons, but the ectodomain of the protein may function as a platform for protein-protein interactions similar to the Ig-like domain of α-sarcoglycan. In fact many mutations linked to MDS are located within the Ig-like domain of ε-sarcoglycan, suggesting it to be key to MDS pathology [17,18]. It was previously shown that most mutations prevent trafficking of the mutated protein to the cell membrane and instead target the protein for endoplasmic-reticulum-associated degradation [19]. However, recently a mutation resulting in a gain of glycosylation was reported which reduces, but not completely abolishes, membrane trafficking of the mutant protein [20]. This mutation also leads to reduced ectodomain- shedding, a process regulating interactions between the cell surface and extracellular matrix. Furthermore, the mutated protein was found to be more susceptible to lysosomal proteolysis than the wild-type ε-sarcoglycan. 

One study of *SGCE K*nock-out mice found the mice carried the paternally-expressed mutated *Sgce* gene and showed a subtle phenotype resembling MDS, including spontaneous myoclonus, motor coordination deficits and potentially impaired motor learning [21]. In the cerebellum, a structure characterised by high ε-sarcoglycan content in wild-type mice, abnormal nuclear envelopes were observed in the Purkinje cells of mutant mice. This is reminiscent of DYT1 dystonia where nuclear envelope abnormalities have been described [22]. To further delineate the role of ε-sarcoglycan in the cerebellum the authors have generated an inducible Purkinje-cell specific Sgce knock-out mouse model. These conditional knock-outs showed impaired motor learning but no myoclonus, coordination deficits or nuclear envelope abnormalities. This suggests a direct role of ε-sarcoglycan in Purkinje cells in motor learning but, loss of this protein in other brain regions, or at an earlier stage in development is required to cause motor deficits, myoclonus and nuclear envelope abnormalities. Another study provided neurophysiological evidence for impaired cerebellar motor learning in MDS patients by testing saccadic adaptation [23]. In comparison to healthy controls, MDS patients had reduced and slower saccadic adaptation indicative of reduced cerebellar motor learning. Further study of the Sgce knock-out mice [24] detected abnormal nuclear envelopes in striatal medium spiny neurons. Conditional knock-out of Sgce specifically in the striatum yielded no myoclonus, nuclear envelope abnormalities or motor learning impairment but mice did exhibit motor deficits. 

These findings suggest that multiple brain regions are involved in bringing about the disease phenotype and, in keeping with studies in primary dystonia, implicate the cerebellum as an important structure. Finally, Beukers *et al*. [25] demonstrated that the severity of dystonia in MDS patients correlated with increased grey matter volumes in the putamen, highlighting the importance of this structure in MDS.

## RAPID-ONSET DYSTONIA PARKINSONISM (RDP; DYT12)

### Clinical Phenotype

RDP is characterized by the abrupt onset of dystonia and parkinsonism and is caused by mutations in the α3 subunit of the Na+/K+ ATPase gene [26,27]. It is inherited as an autosomal dominant trait with variable penetrance, and typically presents abruptly in the second or third decades with an abrupt onset of dystonic spasms, bradykinesia, postural instability, dysarthria, and dysphagia developing over hours to weeks followed by little progression [26,28]. Mild limb dystonia can precede onset by a number of years. Many patients report specific triggers consisting of either physical (fever, running, childbirth, excessive alcohol ingestion) or psychological stress. Limited pathological studies have not identified neurodegeneration suggesting that RDP results from neuronal dysfunction [29]. The symptoms appear to develop with a rostrocaudal gradient, with bulbar symptoms more severe than limb symptoms. This is opposite to the pattern found in other early onset dystonia syndromes such as DYT1-dystonia and (DRD), both of which present with more severe leg involvement. 

### Molecular Pathogenesis

The ATP1A3 gene mutated in RDP encodes the α3 subunit of the Na+/K+ ATPase or “sodium pump”. The Na+/K+ ATPase belongs to the group of P-type ATPases, which utilise energy liberated during the hydrolysis of ATP for active transport of cations across cell membranes. The enzyme is composed of a catalytic α subunit (of which isoforms 1, 2 and 3 are expressed in the nervous system) and an accessory β subunit (of which three isoforms are expressed in the brain) and is embedded in the cell membrane. Isoforms of the Na+/K+ ATPase are expressed in virtually all mammalian cells where they play essential roles in the maintenance of ion gradients across cell membranes. The α1 “housekeeping” isozyme is expressed in most mammalian cells and additionally cells may express cell-type specific isoforms, sometimes confined to different regions in the cell [30]. In the brain, the α3 subunit mutated in RDP is expressed mainly in neurons. Different isozymes of the ATPase have different affinities for ATP, Na^+^ and K^+^ so it is likely that the variation in the relative levels of specific isozymes may be tailored to specific metabolic requirements of a certain cell type. As the activity of the ATPase determines the membrane potential, the predominant expression of one isoform in a specific type of neuron may impact on its electrical properties. For example, the resting potential in hippocampal interneurons and pyramidal cells in the mouse is different, which can be linked to the preferential regulation of ion homeostasis by α3 and α1 isozymes in these cell types respectively [31]. The α3- containing isozyme mutated in RDP has a relatively high affinity for ATP and low affinity for Na^+^ in comparison to the other isoforms. This suggests that during periods of rest, neurons may use the other two isoforms to maintain basal ion homeostasis and recruit the α3-isoforms during high levels of activity. This may explain why defects in the ATP1A3 become only noticeable after stressful events, when the “safety by redundancy” mechanism inherent in the combinatorial expression of different isozymes breaks down. The sudden onset of RDP following heavy exercise or other physical or psychological stress would be consistent with this. 

Biochemical enzyme assays have revealed that mutations in α3 cause a reduction in both Na+ affinity and extrusion of intracellular Na+ leading to disrupted electrochemical ionic gradients across the neuronal cell membrane. Simple organisms such as Drosophila melanogaster provide an easily-accessible and genetically tractable system for the modelling of genetic diseases and random mutagenesis of the fly equivalent of the human ATP1A3 was used to induce an RDP phenotype in Drosophila [32]. All mutations resulted in a loss of function of the ATPase which could be phenocopied by the selective inhibition of the α3-ATPase protein by ouabain and some degree of stress-inducible motor impairment. Recently, Atp1a3 mutant mice, carrying a point mutation immediately upstream of exon 4 leading to aberrant splicing and a dysfunctional α3 subunit, were evaluated as a potential etiologic model of RDP [33]. Interestingly, no difference between wild type and mutant mice was observed in the unstressed state, and after restraint stress only females showed a discernible motor impairment while levels of dopamine and serotonin remained normal. 

A phenotypic mouse model of RDP was recently generated by chemically inhibiting the α3-isoform of the ATPase function in selected brain regions using the targeted infusion of ouabain, which selectively reduces α3-ATPase function [34]. Ouabain infusions in the basal ganglia and cerebellum induced a parkinsonism-like or dystonic-like phenotype, respectively but only concomitant infusions in both structures yielded a stress-inducible phenotype resembling features of RDP. In the mouse model dystonic postures were reduced following transient inhibition of cerebellar input by GABA injection underlining the emerging importance of cerebellar dysfunction in dystonia and RDP. 

## DYSTONIA- PARKINSONISM (DYT16)

### Clinical Phenotype

Camargos *et al* described DYT16 dystonia as a novel, autosomal recessive form of early-onset generalised dystonia, due to putative mutations in the PRKRA gene [35]. The homozygous (P222L) mutation was found in seven affected members from three Brazilian families. All patients were refractory to pharmacological therapy with levodopa and high-dose anticholinergics. The same group also identified two phenotypes, pure generalised dystonia and dystonia-parkinsonism unresponsive to L-dopa [36]. There was marked phenotypic heterogeneity associated with *PRKRA *mutations even within the same family. The patients presented with a gradual onset of limb dystonia in childhood, with prominent, relatively rapid onset, bulbar, cranial-cervical and axial dystonia. Laryngeal dystonia, sometimes leading to anarthria, was a prominent clinical feature, as was the presence of a “sardonic smile”. Parkinsonism appeared later than dystonia in all cases [35].

In one family, the mother of a homozygous carrier was also affected but could not be genotyped. This raised the question of whether a heterozygous mutation might act as a susceptibility factor for dystonia. To explore further the role of mutations in *PRKRA*, Seibler *et al*. screened patients with dystonia and controls for changes in the sequence of this gene. They identified a novel heterozygous mutation (c.266_267delAT; p.H89fsX20) in exon 3 of *PRKRA *in one patient with early onset generalised dystonia [37]. This predicted a frameshift mutation in DYT16 gene, with premature truncation of the protein, and was absent in the other patients and controls. They did not detect any other mutations in *PRKRA *in this patient. The clinical picture was of early-childhood-onset leg dystonia that spread gradually over the course of a few years. Family history was unremarkable, secondary causes of dystonia were excluded, and brain MRI was normal. In addition to the frameshift mutation, Seibler *et al* found two new silent changes (c.126C/T and c.795C/T) in *PRKRA *in two other patients [37]. It remains to be proved whether any of the three sequence changes detected are really pathogenic and whether the patient described is part of the DYT16 spectrum. 

DYT16 cases that present with pure dystonia are an important differential diagnosis of DYT1 and DYT6 dystonia [38]. There are many similarities between *DYT16 *and *DYT12*, such as prominent bulbar signs and rostro-caudal gradient; however, *DYT12 *has an abrupt onset and a clear autosomal dominant mode of inheritance [39].

PKR is an interferon-inducible, double-stranded RNA-activated protein kinase. This protein seems to have an important role in apoptosis pathway from neurons exposed to cell stress, and it is well known that it plays an important role in control of cell death [40]. Drosophila lacking functional PKR have a severe defect in nervous system coordination or neuromuscular function resulting in significantly reduced locomotion [41]. There are recent studies suggesting that PKR is implicated in Alzheimer's disease, [42] extrastriatal degeneration in Parkinson's and Huntington disease [40]. It has also been shown that PKR may also be implicated in neuronal death in prion diseases since phosphorylated PKR-positive neurons demonstrate apoptosis and neuronal degeneration in prion disease [43].

### Molecular Pathogenesis

Mutations in the stress-response gene protein kinase, interferon-inducible double-stranded RNA dependent activator (*PACT) *are associated with *DYT16* dystonia -parkinsonism. PACT is an activator of PKR (protein kinase, RNA activated), an interferon- induced serine-threonine kinase involved in the cellular response to viral infection and stress. Upon activation, PKR changes its conformation resulting in dimerization and autophosphorylation [44]. Double-stranded RNA (dsRNA) produced during viral replication is the most potent activator of PKR but the dsRNA binding motifs of PKR can also mediate protein-protein interactions e.g. with PACT leading to PKR activation in the absence of dsRNA [45]. PACT consists of two redundant PKR binding domains and one distinct PKR activation domain [46] and is itself regulated by TAR DNA binding protein (TRBP). In the unstressed state, TRBP sequesters PACT and so prevents it from activating PKR. Under stress, PACT is released from TRBP [47], allowing it to stably dimerize [48] and bind PKR thereby facilitating PKR autophosphorylation and catalytic activation [49]. The exact mechanism by which the proven and putative mutations linked to DYT16 cause the observed clinical phenotype is unclear. The P222L mutation lies between a binding and the activator domain in PACT and may interrupt PACT-PKR interaction due to reduced binding capability of misfolded PACT or loss of function in the activator domain. Mutations abolishing correct functioning of the activation domain render PACT inhibitory to PKR [50]. Likewise, a truncated protein resulting from the possible frameshift mutation may have a dysfunctional or even absent activation domain. It is likely that the reduced function of the PKR-mediated stress response pathway in DYT16 leaves these cells more susceptible to stress.

## HEREDODEGENERATIVE DYSTONIAS

Heredodegenerative disorders are those where dystonia is part of a more complex phenotype due to underlying neurodegeneration. They are best classified by aetiology: disorders of metabolism, mitochondrial diseases, trinucleotide repeat disorders, parkinsonian disorders, and other degenerative processes without defined cause. A classification of these disorders is shown in Table **2**. Neurodegeneration with brain iron accumulation is described in a separate review. Here we describe those conditions where there have been advances in understanding of the molecular pathogenesis in recent years. 

## LESCH-NYHAN DISEASE 

### Clinical Phenotype

In 1964, Lesch and Nyhan described 2 brothers with a clinical syndrome characterized by hyperuricemia, hyperuricosuria and severe neurologic dysfunction including choreoathetosis, mental retardation, and self-injurious behaviour [51]. Lesch-Nyhan disease (LND) is an X-linked disorder resulting from deficiency of hypoxanthine–guanine phosphoribosyltransferase (HPRT). Clinical manifestations include movement disorders such as dystonia, chorea, spasticity, self-mutilation, hyperuricemia and developmental delay. The diagnosis is suggested by elevated levels of uric acid in serum and urine, and secured by absent HPRT activity in cultured fibroblasts.

Early features of HPRT deficiency relate to uric acid overproduction and one of the first signs of the disease may be the observation of orange crystals in the diapers, or crystalluria with obstruction of the urinary tract. Psychomotor delay, when present, becomes evident within 3 to 6 months. A delay in the acquisition of sitting and head support with hypotonia and athetoid movements may lead to neurological consultation. Self-mutilation, in the form of lip biting or finger chewing, can appear as soon as teeth are present.

The spectrum of clinical features associated with HPRT deficiency is broad. Although complete HPRT deficiency typically results in the typical LND syndrome, partial deficiency more often causes a phenotype in which some features are attenuated or absent [52,53]. Collectively, these patients are labelled LND variants. All of these patients produce excess uric acid, but have variable neurological and behavioural features. 

Complete deficiency of HPRT activity causes classic LND. Affected individuals typically suffer from hyperuricaemia, nephrolithiasis, gout or subcutaneous deposits of tophi [52]. Neurologically, all patients have severe motor dysfunction that is dominated by dystonia, with additional pyramidal features (spasticity, hyperreflexia and clonus) [54]. Self-injurious behaviour, including biting and head banging is common often accompanied by self-mutilation, impulsivity, striking or spitting at others, or use of socially unacceptable language [55-57]. Most have cognitive decline [58,59] with significant reduction in global IQ, compounded by inattention.

Despite the spectrum of problems related to uric acid, it seems unlikely that they are causally related to the neurological or behavioural problems in LND. Treatment of LND patients from birth with allopurinol does not influence the development of neurobehavioural problem. In addition, there are other clinical disorders with excessive production of uric acid without the neurobehavioural problems of LND [52].

### Molecular Pathogenesis

In LND patients, HPRT activity is generally absent or below 1.5% of normal [60]. Over 2000 mutations have been described [61] which reduce or abolish HPRT function by several mechanisms including aberrant protein folding and reduced substrate affinity or enzyme synthesis. There is evidence of degeneration of dopaminergic midbrain neurons LND [62] which is likely to be the cause of the dystonic motor phenotype. However, the exact pathogenesis by which disrupted purine metabolism may cause death of mesencephalic dopaminergic and other neurons is not known. 

In one study, HPRT deficient murine neuroblastoma cells were differentiated into dopaminergic neurons which exhibited reduced neurite outgrowth [63]. Quantitative PCR analysis in these cells showed a significant over-expression of the transcription factors engrailed 1 and 2. This appeared to be directly related to HPRT deficiency, as engrailed levels normalised after HPRT activity had been restored. Engrailed proteins act as axon guidance factors, regulating mRNA translation in growth cones [64], and en1 has been detected in dendrites of dopaminergic neurons [65]. Abnormal neurite outgrowth was also observed in a recent study that used human neural stem cells (NSC) carrying LND mutations to generate dopaminergic neurons *in vitro* [66]. HPRT-deficient NSCs were found to exhibit reduced neurogenesis even after only 3 days of differentiation. Dopaminergic neurons derived from LND NSCs had significantly reduced neurite length as compared to healthy controls. Furthermore, quantitative PCR analysis revealed a 30-fold reduction of aldehyde dehydrogenase (ALDH1) expression in LND NSCs. ALDH1 is essential to the biosynthesis of retinoic acid (RA), a molecule which has been implicated in axon guidance. To establish the causality of ALDH1 under-expression in the generation of morphologically aberrant dopaminergic neurons from HPRT-deficient NSCs, the authors tested whether supplementation of culture medium with RA during the differentiation period could rescue the phenotype. This was found to be the case, suggesting reduced RA levels contribute to the pathology and phenotype of LND *in vitro*.

## DOPAMINE TRASNPORTER GENE MUTATIONS 

### Clinical Phenotype

Dopamine transporter deficiency syndrome is the first identified movement disorder caused by genetic alterations of the dopamine transporter [67,68]. Children presented in infancy with either hyperkinesia, parkinsonism, or a mixed hyperkinetic and hypokinetic movement disorder. Some individuals had previously been misdiagnosed with cerebral palsy. During childhood they developed severe dystonia parkinsonism associated with an eye movement disorder and pyramidal tract features. There were no associated psychiatric or cognitive features. 

Investigations revealed raised ratios of homovanillic acid to 5-hydroxyindoleacetic acid in cerebrospinal fluid. DAT scan imaging in one patient showed complete loss of dopamine transporter activity in the basal nuclei.

Although a trial of L-dopa had no effect on either the patients’ symptoms, or on CSF parameters, there was moderate improvement following deep brain stimulation in one case [67].

### Molecular Pathogenesis

Deficiencies in Dopamine Transporter (DAT) expression is an autosomal recessive disorder. DAT is a transmembrane protein exclusively expressed in dopaminergic neurons where it mediates the reuptake of dopamine into pre-synaptic terminals after synaptic transmission. This rapid recycling of neurotransmitter is crucial to synaptic function as it replenishes dopamine stores in the presynaptic terminal and prevents desensitization of the postsynaptic terminal.

To investigate the mechanisms by which mutations in the *DAT* gene impair DAT protein function, [67] a study expressed the mutated *DAT* genes from 11 patients in HEK 293 cells. All mutations produced loss of DAT function, although the mechanisms by which this was brought about were diverse and included reduced gene expression, decreased dopamine binding affinity, impaired protein maturation and aberrant trafficking of DAT to the cell membrane.

Study of mutant DAT mice [68] revealed increased perisynaptic levels of dopamine resulting from reduced re-uptake has both pre- and postsynaptic implications. At the presynaptic terminal, dopamine stores were not replenished after neurotransmission leading to reduced dopamine pools. Furthermore dopamine may act on presynaptic D3 receptors leading to downregulation of TH activity and hence dopamine biosynthesis. Postsynaptically, desensitisation or internalisation of dopamine receptors may further contribute to reduced efficiency of the dopaminergic synapse.

## LEBER HEREDITARY OPTIC NEUROPATHY (LHON, MITOCHONDRIAL)

### Clinical Phenotype

Mutations in mitochondrial respiratory chain complexes I, III or IV may lead to Leber’s hereditary optic neuropathy, a disorder characterized by rapid, often sequential, visual loss beginning between ages 18 and 23 [69]. There is bilateral painless optic atrophy due to death of retinal ganglion cells. Dystonia, ataxia or spastic paraplegia may accompany the visual changes (Nikoskelainen *et al* 1995). Transmission is maternal with reduced penetrance of <50% in males and <20% in females. Diagnosis is supported by an elevated serum lactate and microangiopathic changes in the optic fundus. Direct DNA mutation analysis is available. Autosomal dominant optic neuropathy due to mutations in the *OPA1*gene has also been described.

### Molecular Pathogenesis

There are three main pathogenic mutations causing LHON (11778/ND4, 3460/ND1,14484/ND6) all disrupting components of complex 1 of the mitochondrial respiratory chain [70]. The malfunction of mitochondrial oxidative phosphorylation may cause neuronal degeneration by energy depletion and generation of reactive oxygen species (ROS). In most cases pathology is limited to the optic nerve, although additional features including dystonia or other motor disorders have been described. One of the puzzling facts about LHON is its incomplete penetrance, even when patients are homoplasmic for the mutation (100% mitochondrial genomes are mutant in each cell), and the increased penetrance observed in males. Around 30-50% of male and 10-20% of female carriers actually manifest with the disease, suggesting the LHON mutation to be necessary but not sufficient to induce the disease. It has been suggested that multiple other genetic and environmental factors account for this variability in penetrance and gender preference. Recently Giordano *et al*. [71] investigated the effects of oestrogens on cybrids carrying the LHON mutations 11778/ND4, 3460/ND1 and 14484/ND6 *in vitro*. Treatment with 17β-oestradiol reduced levels of reactive oxygen species (ROS), believed to be responsible for oxidative cell damage which had previously been shown to be raised in LHON cells. This reduction in ROS appeared to be caused by a direct induction of superoxide dismutase 2 (SOD2), the main mitochondrial antioxidant enzyme. Furthermore, oestradiol treatment was shown to improve the cellular energetic competence probably by reducing mitochondrial network fragmentation and increasing mitochondrial biogenesis. This enhanced cellular fitness manifested itself in improved cell viability and reduced apoptosis. The observed effects were shown to be oestrogen-dependent as chemical inhibition of the oestrogen receptor β abolished all beneficial effects. In humans, the oestrogen receptor β is present in the mitochondria of retinal ganglion cells and the unmyelinated portion of the axons of surviving optic fibres. This demonstrates the relevance of the findings obtained *in vitro* to the disease in humans and suggests a metabolic basis for the observed increased prevalence in males.

The observed reduced penetrance could be due a compensatory upregulation of mitochondrial density in non-symptomatic carriers and late-onset–onset patients. Korsten *et al*. [72] demonstrated that while complex 1 and 2 of the mitochondrial electron transport chain exhibited reduced ATP-production rates in manifesting and non-manifesting patients with mutations m.3460G>A (*n* = 5), m.11778G>A (*n* = 8), and 14484T>C (*n* = 4), non-manifesting carriers had normal ATP-production *per cell*. All LHON mutation carriers had increased mitochondrial densities per cell, but only in non-manifesting patients was this sufficient to raise ATP production to normal levels *per cell* in spite of reduced ATP production* per mitochondrion*. This study provided a biochemical phenotype in peripheral blood mononuclear cells which might be useful for prognostic predictions of severity and onset of the disease. 

## DYSTONIA DEAFNESS SYNDROME (MITOCHONDRIAL)

### Clinical Phenotype

Dystonia-Deafness syndrome (DDS, also called Mohr-Tranebjaerg syndrome) is a rare X-linked progressive neurodegenerative disorder. It is caused by mutations in the gene for DDP1, which leads to a defect in the import of carrier protein into the mitochondria and insertion into the mitochondrial inner membrane. DDS was originally described in a family with X-linked progressive sensorineural deafness. Later study of the family identified additional clinical features, including progressive dystonia, spasticity, mental deterioration, and blindness. Sensorineural deafness is an invariable feature, typically with onset soon after language milestones are reached (postlingual deafness). Dystonia is also an almost universal feature of cases with *DDP1 *mutations with onset age between 6 and 30 years. Focal dystonia is described, but usually the dystonia associated with *DDP1 *mutations is generalized with involvement of craniocervical muscles. The dystonia is progressive and other motor features such as spasticity develop. 

### Molecular Pathogenesis

Dystonia-Deafness is caused by mutations in the *TIMM8A* gene which encodes the mitochondrial protein dystonia deafness protein 1 (DDP1), whose role in mitochondrial function and biogenesis is poorly understood. A recent study tested the effects of elevated or reduced DDP1 levels on mitochondrial morphology, energy production, cellular viability and protein import [73]. Bioenergetic properties were unchanged in patient fibroblasts and DDP1 knock-down cells compared to controls, but the import of beta-barrel proteins and mitochondrial morphology was abnormal. Reduced DDP1 expression yielded long thread-like mitochondria, whereas overexpression produced hollow grain-like mitochondria. It was suggested that reduced import of beta-barrel proteins into the intermembrane space (IMS) resulted from mutant DDP1 failing to form import complexes and accumulation of monomeric forms in the IMS. Reduced DDP1 levels did not alter the bioenergetic properties (membrane potential of the affected mitochondria or oxygen consumption), arguing against a energetic deficit as the main cause of the disease. These findings suggest that, although mutations in DDP1may not cause a direct metabolic deficit in the tested fibroblasts, altered mitochondrial morphology may disrupt mitochondrial transport and function in neurons.

## LEIGH SYNDROME

### Clinical Phenotype

Leigh syndrome (LS) is a genetically heterogeneous disease that usually presents in infancy with subacute necrotising encephalopathy [74]. Onset is typically in infancy or childhood, with developmental regression, seizures, brainstem dysfunction, dystonia, ataxia and optic atrophy. The diagnosis is supported by finding elevated levels of lactate and pyruvate in serum and cerebrospinal fluid, abnormal pattern of cyctochrome c oxidase expression on muscle biopsy, and abnormal MRI with evidence of necrosis in the basal ganglia, thalamus and brainstem. Other manifestations include failure to thrive, microcephaly, hypertrichosis, and myopathy [75,76]. Progressive disturbances of the respiratory function due to necrotizing brainstem lesions are often fatal.

### Molecular Pathogenesis

LS typically occurs as the result of mutations in the gene encoding SURF-1, important for cytochrome c oxidase assembly [77]. Other mitochondrial enzymatic abnormalities have been described, including defects in pyruvate dehydrogenase and respiratory complexes I, II, IV and V [78]. Leigh syndrome may be inherited in a maternal, X-linked or autosomal recessive fashion. LS is associated with a systemic deficiency of components of the mitochondrial electron transport chain. In most cases mutations disrupt the biogenesis of the complexes, although a recent study demonstrated that catalytic impairment of complex I is sufficient to cause the disease without any reduction in overall protein levels [79].

In an attempt to delineate the cellular pathogenesis of LS, Verkaart *et al*. [80] investigated patient fibroblasts lines carrying various LS mutations resulting in reduced complex I levels. They found that increased ROS production was inversely proportional to complex I levels. The mitochondrial membrane potential, ΔΨ, was found to be depolarised in LS mitochondria compared to healthy controls and depolarisation was directly proportional to ROS production. Both depolarisation and ROS production are indicators of poor mitochondrial health. Mitochondrial morphology was also affected: Severe complex I deficiency lead to fragmented mitochondria but less severe deficiency had no effect suggesting there to be a certain threshold-level rather than a linear relationship.

Mutations in the *Surf1* gene lead to deficiency of cytochrome oxidase (COX), complex IV in the mitochondrial electron transport chain. *Surf1* protein is involved in the assembly of COX and mutant protein leads to reduced levels of correctly assembled and fully functional COX. 

A recent study used a decerebrate *Surf1* KnockOut mouse model to investigate aberrant neural activity in brainstem breathing centres [81]. While minimal respiratory abnormalities were observed at rest, under hypoxic conditions mutant mice showed highly irregular respiratory activity and augmented breaths/sighs. This elevated respiratory frequency and pattern irregularity may be due to an impaired function of arterial oxygen sensing chemoreceptors. This would support previous work that COX function in the mitochondria of carotid and aortic bodies is necessary for their chemosensing properties [82]. If these findings obtained in the mouse model apply to human patients, this may direct efforts to develop new therapeutic strategies towards treating early-stage respiratory abnormalities and highlights the need for preventive measures ensuring patients do not experience hypoxia.

## DISCUSSION

This review demonstrates the rapid increase in our knowledge of the cellular pathogenesis of various forms of dystonia plus and heredodegenerative disorders in the last 5 years. Whilst the conditions appear disparate, a number of common themes emerge which recapitulate studies in primary dystonia:

### 1 Abnormalities in Dopaminergic Neurotransmission

At the molecular and cellular level there is increasing evidence for abnormalities in dopaminergic neurotransmission in dystonia. This is demonstrated by DRD, DAT gene related dystonia-parkinsonism and LND. In DYT1 dystonia there is also evidence to support a dopaminergic hypothesis with abnormal synaptic vesicle function [83,84]. Other forms of primary dystonia, including *DYT6*, have been associated with reduced striatal D2 receptor availability, and it has been suggested this may lead to dystonic movements by affecting the indirect pathway of cortical-basal ganglia circuits.

### 2 Oxidative and Endoplasmic Reticulum Stress

There is also evidence for involvement of other cell pathways such as endoplasmic reticulum stress and response and oxidative stress [1,85] which overlaps with findings in MDS and mitochondrial disease. The role of mitochondria in primary dystonia has also been supported by studies of complex I activity in these patients [86]. Impaired capability of cells to cope with stress could leave neuronal cells more vulnerable and prone to misfunction ultimately causing the dystonic phenotype [85].

### 3 Involvement of Cerebellar and Basal Ganglia Pathways

The neuroanatomy underlying dystonic movements reported in this review also highlights the importance of cerebellar pathways, as well as the basal ganglia, which also reflects functional imaging studies in primary dystonia [87]. This suggests that at least some types of dystonia are caused not only by localised dysfunction of the basal ganglia but by the mis-concerted activity of more wide-spread neuronal networks spanning multiple brain regions.

These developments emphasise the need to study these rarer dystonia plus and heredodegenerative disorders, not least to understand their own unique pathogenic mechanisms, but to give greater understanding of the molecular pathogenesis of all forms of dystonia.

## Figures and Tables

**Table 1.** Overview Over Dystonia –Plus Syndromes

Disease	Gene	Functional Defect	Phenotype
DRD/ DYT5a	*GCH* *PTPS * *PCD * *DHPR*	BH4 deficiency, Reduced dopamine biosynthesis, oxidative stress	Dopa-responsive dystonia
DRD/ DYT5b	*TH*	Reduced dopamine biosynthesis	Dopa-responsive dystonia, Cognitive deficits
DYT11	*SGCE*	Unknown, synaptic transmission/adhesion?	Myoclonus-Dystonia
RDP/DYT12	*ATP1A3*	Dysregulated neuronal ion homeostasis	Rapid-onset dystonia parkinsonism
DYT16	*PACT*	Unknown	Early-onset dystonia parkinsonism

Table **1** Abbreviations: DRD Dopa-responsive dystonia, GCH GTP- cyclohydrolase 1, PTPS 6-pyruvoyltetrahydropterin synthase, PCD pterin-4α-carbinolamine dehydratase, DHPR dihydropteridine reductase, TH tyrosine hydroxylase, SGCE epsilon-sarcoglycan, RDP Rapid-onset dystonia parkinsonism, ATP1A3 alpha3- Na+-K+-ATPase, PRKRA Protein kinase interferon-inducible double stranded RNA dependent activator.

**Table 2.** Heredodegenerative Disorders with Dystonia

**Metabolic Disorders**	Metal and Mineral	Wilson’s disease, Neurodegeneration with brain iron accumulation 1, Neuroferritinopathy, Idiopathic basal ganglia calcification (Fahr disease)
	Lysosomal Storage Disorders	Niemann Pick C, GM1 and GM2 gangliosidosis, metachromatic leukodystrophy, Krabbe disease, Pelizaeus-Merzbacher disease, fucosidosis, neuronal ceroid lipofuscinosis,
	In-born errors of metabolism	Lesch-Nyhan syndrome, Triosephosphate Isomerase deficiency, Aromatic amino acid decarboxylase deficiency Glucose transport defects
	Amino and organic acidurias	Glutaric aciduria type I, Homocystinuria, proprionic aciduria, Methylmalonic aciduria, 4-hydroxybutyric aciduria, 2-oxoglutaric aciduria, Hartnup disease isolvaleric acidemia
**Mitochondrial Disorders**		Leigh’s Syndrome Leber’s Hereditary Optic Neuropathy Human deafness-dystonia syndrome (Mohr-Tranebjaerg syndrome)
**Parkinsonian Disorders**		Parkinson’s disease, Progressive supranuclear palsy Corticobasal degeneration Juvenile-onset parkinsonism X-linked dystonia-parkinsonism Rapid-onset dystonia-parkinsonism
**Triple Repeat Disorders**		Huntington’s disease Spinocerebellar ataxia
**Other**		Ataxia telangectasia, Chorea-acanthocytosis, Rett syndrome, Infantile bilateral striatal necrosis, neuronal intranuclear disease Ataxia with vitamin E deficiency, Progressive pallidal degeneration, Sjogren-Larsson syndrome, Ataxia-Amyotrophy-Mental-retardation-Dystonia syndrome
